# F218. REAL-TIME ASSESSMENT OF AUDITORY HALLUCINATIONS USING A SMARTPHONE APPLICATION; RESULTS FROM A PILOT STUDY

**DOI:** 10.1093/schbul/sby017.749

**Published:** 2018-04-01

**Authors:** Josef Bless, Runar Smelror, Ingrid Agartz, Kenneth Hugdahl

**Affiliations:** 1 University of Bergen; 2 Norwegian Center of Excellence for Mental Disorders Research, University of Oslo, Diakonhjemmet Hospital; 3 University of Bergen, Norwegian Center of Excellence for Mental Disorders Research, University of Oslo, Haukeland University Hospital

## Abstract

**Background:**

A challenge in current research on auditory hallucinations (AHs) is that the assessment of symptom dimensions largely depends on structured interview scales, such as the PANSS, PSYRATS etc. In order to collect more ecologically valid data, we developed a smartphone app that can be used by patients to report on their experience in real-time, i.e. when the voices are actually present. The aim of this study was to investigate feasibility of the app and whether it can provide new phenomenological information on the temporal fluctuations of AHs in adolescent patients with early-onset schizophrenia (EOS).

**Methods:**

Using the experience sampling method, one adolescent EOS patient used the app for a period of 16 days, during which the patient received random reminders five times per day, to answer questions on five dimensions relevant to AHs: Control (no – full), Content (negative – positive), Localization (outside head – inside head), Intensity (yelling – whispering), and Influence (not troublesome – very troublesome). The answers were registered on visual analog scales (VASs) implemented in the app.

**Results:**

The patient responded to the notifications in 87% of the cases and in addition completed the questions 15 times on own initiative. In 73% of all responses, the patient indicated to experience AHs at the time of response. The results from the VASs showed that AH-dimensions are not stable but fluctuate over time. Several AVH-dimensions were significantly correlated (p < .01) with each other: Influence correlated with Content (r = -.71), Intensity (r = -.37), and Control (r = -.76), whereas Content correlated with Intensity (r = .39) and Control (r = .57). showed several correlations a negative correlation with content of however, only localization (voices coming from outside - inside the head) correlated significantly with the number of days in use. In addition, the participant reported more internal voices over the course of 16 days (p < .01; r = .36) and later hours of the day (p < .05; r = .22).

**Discussion:**

The app captures the ebb-and-flow of AVHs and provides a unique profile of symptom severity and interrelationship between AVH-dimensions. Such information has potential relevance for patient-tailored intervention.

